# Demonstration of the spin solar cell and spin photodiode effect

**DOI:** 10.1038/ncomms3068

**Published:** 2013-07-03

**Authors:** B. Endres, M. Ciorga, M. Schmid, M. Utz, D. Bougeard, D. Weiss, G. Bayreuther, C.H. Back

**Affiliations:** 1 Institut für Experimentelle und Angewandte Physik, Universität Regensburg, Regensburg 93053, Germany

## Abstract

Spin injection and extraction are at the core of semiconductor spintronics. Electrical injection is one method of choice for the creation of a sizeable spin polarization in a semiconductor, requiring especially tailored tunnel or Schottky barriers. Alternatively, optical orientation can be used to generate spins in semiconductors with significant spin-orbit interaction, if optical selection rules are obeyed, typically by using circularly polarized light at a well-defined wavelength. Here we introduce a novel concept for spin injection/extraction that combines the principle of a solar cell with the creation of spin accumulation. We demonstrate that efficient optical spin injection can be achieved with unpolarized light by illuminating a p-n junction where the p-type region consists of a ferromagnet. The discovered mechanism opens the window for the optical generation of a sizeable spin accumulation also in semiconductors without direct band gap such as Si or Ge.

## Abstract (web summary)

Finding ways to accumulate electronic spins of a given polarization in a given location is important to the development of spintronics. Endres *et al.* demonstrate a device that uses light to drive the accumulation of spin using a similar principle that a solar cell uses to drive the accumulation of charge.

## Graphical abstract

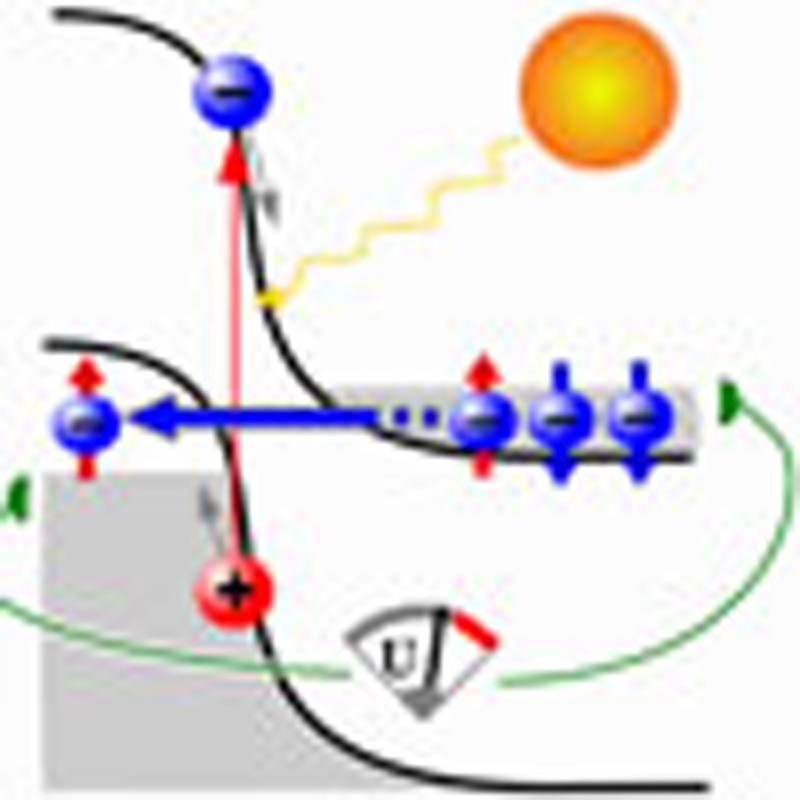

One of the main quests in spintronics research today is the efficient and energy conserving generation of pure spin currents. Ideally, and mainly in order to reduce energy consumption, pure spin currents should be generated without the use of charge currents that cause Joule heating. Modern concepts avoiding charge currents in the conventional sense range from spin pumping^1^,^2^,^3^ to thermal injection^4^, while electrical spin injection^5^,^6^ using a direct or non-local (NL) approach requires the use of charge currents. In semiconductors, optical spin pumping is also a convenient way to generate spin-polarized carriers without the need of charge currents^7^. However, owing to the optical selection rules this method usually requires circularly polarized light at a well-defined wavelength.

New effects can be expected when instead of a homogeneous semiconductor a p-n junction is illuminated. A spin-voltaic effect has been predicted for a magnetic/non-magnetic p-n junction, where either the p-type or the n-type semiconductor is magnetic, that is, has a spin-split band^8^,^9^. This effect has been experimentally demonstrated in a non-magnetic n-GaAlAs/p-GaInAs/p-GaAs junction in the presence of a magnetic field allowing to convert circularly polarized light into an electric signal^10^. More recently the feasibility of a ‘spin-photodiode’ has been demonstrated^11^ where a spin-polarized current is produced in a Fe/MgO/Ge heterostructure by absorption of circularly polarized light. Here, we demonstrate a spin-generating solar cell comprising a p-n junction, where the p-side of the junction is a ferromagnetic semiconductor that enables efficient optical spin injection with unpolarized light.

Figure 1a shows the energy diagram of a p-n junction based on GaAs. When illuminated by photons with an energy exceeding the band gap, electron-hole pairs are generated. These pairs are separated in the built-in electric field of the p-n junction and give rise to a photo-voltage (photo-current). In the device presented here, we employ a ferromagnetic version of a solar cell by using the ferromagnetic semiconductor (Ga,Mn)As (refs 12, 13) on the p-side. Together with a highly doped n-side this results in a narrow depletion zone and enables tunnelling across the gap. For sufficiently high doping the *I–V* characteristic becomes Esaki diode like^14^. Owing to the heavily p-doped (Ga,Mn)As, the band bending region is mostly confined to the n-GaAs. If such a p-n junction is illuminated, the resulting photo-current will mostly consist of photoexcited electrons from the n-GaAs side and only a small fraction of spin-polarized electrons is created in the (Ga,Mn)As. Thus, the spin polarization of the photo-current is small. The charge accumulation in the n-GaAs leads to a photo-voltage which in turn causes electrons to tunnel across the narrow barrier into the (Ga,Mn)As. Owing to the different tunnelling probabilities for spin-up and spin-down electrons, spins accumulate in the n-GaAs, that is, light-induced spin extraction occurs (see Fig. 1b) that overcompensates the photo-current-induced spin accumulation. This constitutes the central working principle of our spin solar cell: the energy of the incident light is not only converted into a voltage (current) but also into a spin accumulation.

In not too highly n-doped junctions where tunnelling is suppressed in reverse direction another mode of operation, sketched in Fig. 1c, becomes possible. Applying a negative voltage to the p-side of the junction (reverse bias) increases the depletion width and suppresses tunnelling. Photoexcited electrons on the p-side are, due to the spin-dependent density of states in the valence band^15^, spin-polarized and drift in the electric field of the junction into the conduction band of n-GaAs. This spin photodiode effect was predicted in 2001 (ref. 16) and also results in a spin accumulation, however, with the spin orientation reversed in comparison to the spin solar cell effect (compare Fig. 1b). This means light-induced spin injection occurs.

In the following, we present the experimental realization of a spin solar cell and a spin photodiode for a diode consisting of a (Ga,Mn)As p-layer and a 1-μm-thick n-GaAs channel with a highly doped interface region (see Methods). We investigate the effect on two samples from two different wafers with different n-doping levels close to the p-n junction (see Methods). In Sample A, both effects are demonstrated by using a laser beam simultaneously to generate electron-hole pairs and to detect spin accumulation via the polar magneto-optic Kerr effect. We employ a cross-sectional two-dimensional imaging method that allows probing the spin polarization also below the ferromagnetic contacts at cryogenic temperatures^17^,^18^,^19^. For the optical detection, a modulation technique is used, so that the Kerr rotation *θ*_K_ is strictly proportional to the spin accumulation 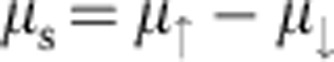 in the n-GaAs channel at the laser spot position (
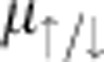
 is the chemical potential for up/down spin electrons). The spin solar cell effect is then additionally demonstrated in Sample B by probing the generated spin accumulation electrically in a NL geometry^6^, where we directly compare light induced with electrical spin injection. The optical detection of the spin photodiode effect is demonstrated in Sample A; here, the sign reversal compared with the solar cell effect confirms the interpretation of the processes sketched in Fig. 1.

## Results

### Electrical spin extraction

We first demonstrate electrical spin extraction in Sample A. Figure 2a shows a linescan of the Kerr rotation *θ*_K_ along the n-GaAs channel for electrical spin extraction, that is, when driving a current between the (Ga,Mn)As and a reference contact. The position of the (Ga,Mn)As contact is indicated by the shaded area. The measurement principle is sketched on the left hand side. On the right hand side of the (Ga,Mn)As contact, due to the application of the bias voltage, the spin density distribution is determined by the superposition of electron drift and spin diffusion opposing each other. On the left hand side of the contact the spin transport is purely diffusive and a spin diffusion length of 6 μm can be extracted from the exponential decay of *θ*_K_.

### Spin solar cell

After removing the electrical connections, the Kerr rotation trace shown in Fig. 2b clearly displays spin accumulation when the laser spot is placed on the (Ga,Mn)As/GaAs interface. The signal immediately vanishes when the spot is moved away from the junction area, indicating that the spin accumulation is induced by laser illumination. In addition a positive photo-voltage *V*_*PV*_ of 130 mV is detected between the (Ga,Mn)As contact and the n-GaAs channel, when the laser spot is located at the contact region (*y*=0 μm in Fig. 2b) (see Supplementary Fig. S1). The inset of Fig. 2b shows the depolarization of the generated spin accumulation in an out-of-plane magnetic field, that is, Hanle measurement^19^. The fit yields a spin lifetime of 13 ns in the n-GaAs channel (for details see Supplementary Note 1). The exclusion of other effects, for example, thermal spin injection due to Joule or laser heating is discussed in Supplementary Fig. S2.

**Figure 1:**
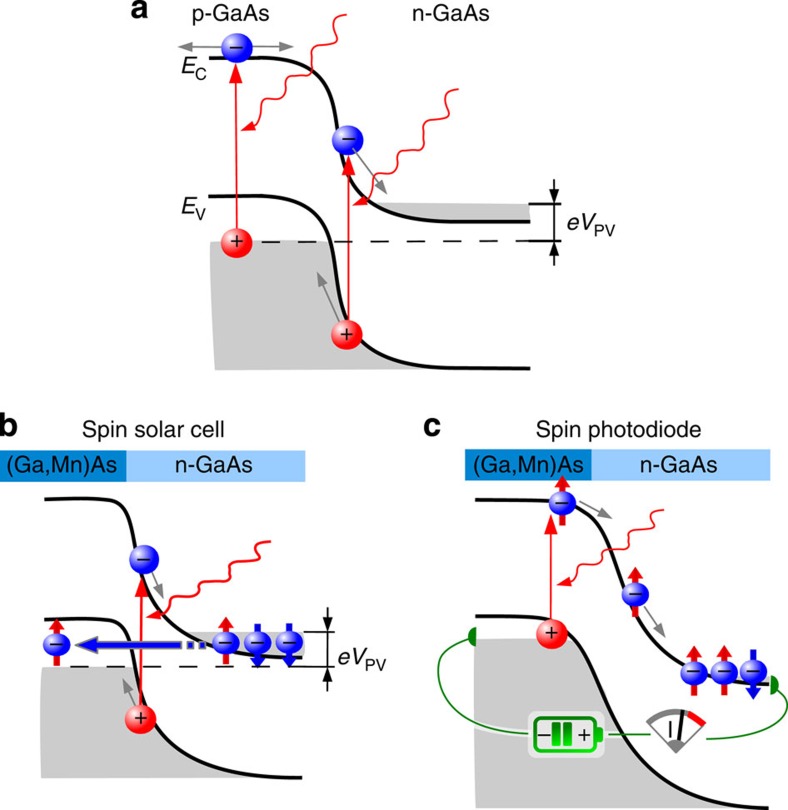
Working principles of spin solar cell and spin photodiode. (**a**) Schematic of an illuminated GaAs p-n junction showing the conduction and valence band edge *E*_C_ and *E*_V_ across the junction. Electron-hole pairs are separated in the electric field of the space charge region (see grey arrows) and generate a photo-voltage *V*_PV_. Grey areas indicate occupied states. (**b**) Working principle of the spin solar cell (open circuit condition): the light-induced photo-voltage drives an electron tunnelling current (blue arrow) across the gap resulting in a spin accumulation on the n-GaAs side. Photoexcited electrons are only weakly polarized. (**c**) Working principle of the spin photodiode (biased circuit condition): at reverse bias the width of the tunnel barrier (depletion zone) increases and tunnelling is suppressed. As a consequence, photoexcited electrons from the (Ga,Mn)As, which are spin-polarized, are drifting to the n-GaAs conduction band and generate an oppositely oriented spin accumulation.

**Figure 2:**
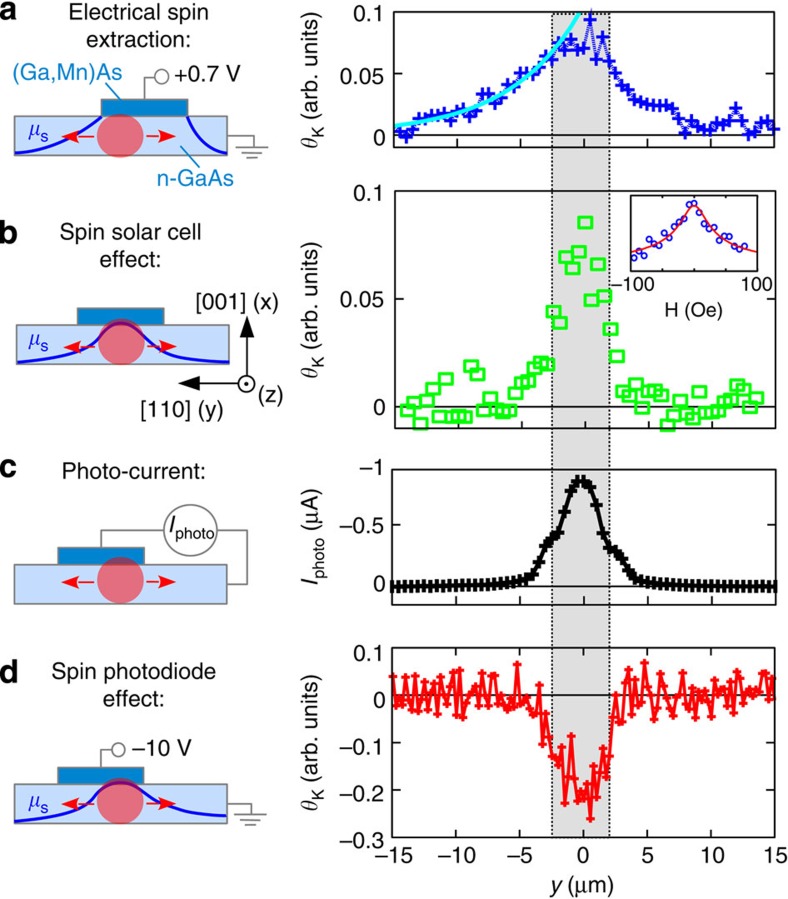
Optical detection on Sample A. Spin accumulation and photo-current along the n-GaAs channel at 15 K. The shaded area in the right panels indicates the position of the (Ga,Mn)As contact; the red circular area in the pictograms on the left represents the laser spot of 1 μm diameter. (**a**) Kerr rotation proportional to the local spin accumulation for electrical spin extraction biased with 0.7 V/12 μA; laser parameters for Kerr detection are *λ*=816 nm and absorbed laser power *P*_abs_=35 μW μm^−2^. A spin diffusion length of 6 μm is deduced from the signal decay in the field-free region left of the contact. (**b**) Kerr rotation as a consequence of the spin solar cell effect using the same laser parameters as in **a**. Spin diffusion also occurs in this situation and would be detected if a separate probe beam were used to observe the spin density distribution created by the spin solar cell effect. The inset shows the Hanle depolarization curve of the spin solar cell effect in an out-of-plane magnetic field; a numerical fit yields a spin lifetime of about 13 ns. (**c**) Position-dependent photo-current measured for the same laser parameters as in **a** and **b**. (**d**) Spin photodiode effect measured for *λ*=816 nm and *P*_abs_=103 μW μm^−2^ at reverse bias of −10 V.

When short-circuiting the (Ga,Mn)As contact and the n-GaAs channel, as sketched in the pictogram of Fig. 2c, a photo-current flows. The close correspondence between this current and *θ*_K_ in Fig. 2b is further evidence of the spin solar cell effect. A negative voltage applied to the (Ga,Mn)As contact, shown in Fig. 2d, reverses the sign of the spin accumulation as expected from the spin photodiode effect (see Fig. 1c). This will be discussed in more detail below.

### Electrical detection

**Figure 3:**
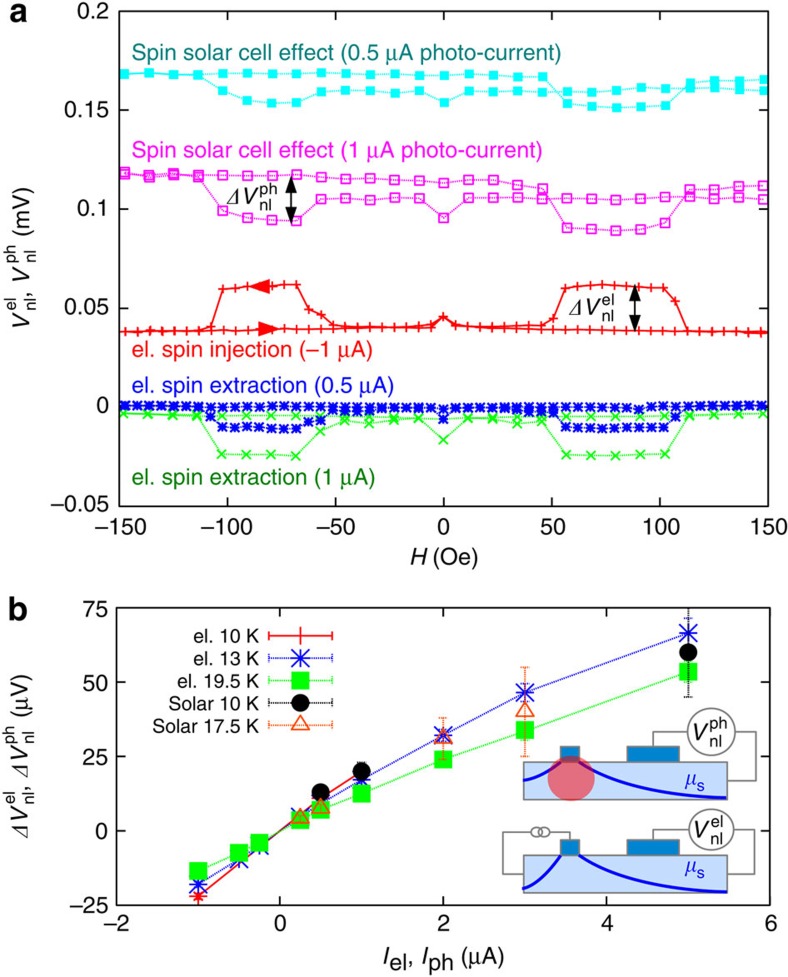
Electrical detection on Sample B. Electrical detection of the optically and electrically created spin accumulation in a NL geometry. (**a**) Observed NL voltage while sweeping the magnetic field along the z direction (spin independent offset voltage subtracted only from spin solar cell curves for clarity: 0.2 and 0.6 mV, respectively. The larger noise level and slight drift are ascribed to illumination and the limited long-term mechanical stability of the optical setup). (**b**) Extracted NL spin valve signal (
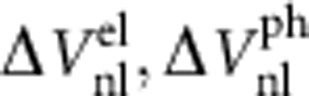
) from the solar cell effect and electrical spin injection versus the applied current or measured photo-current (adjusted by the laser intensity), respectively. Error bars represent absolute minima and maxima. The inset illustrates the measurement geometry for both cases.

To further substantiate the presented spin solar cell mechanism, we probe this effect also electrically in a NL geometry^6^, which allows separation of illumination and detection. The corresponding measurements are performed on Sample B, which has a 0.5-μm wide (Ga,Mn)As injection contact and a 4-μm wide (Ga,Mn)As detector at a distance of 4 μm (see inset in Fig. 3b). The *I–V* characteristic of both contacts is shown in Supplementary Fig. S4. The generated spin accumulation below the narrow contact (spin diffusion length 6 μm, spin lifetime 15–20 ns, see Supplementary Figs S5 and S6) can be observed by measuring the NL voltage signal between the detector and a reference contact placed at a distance of 300 μm. In Fig. 3a, we compare the NL voltage from electrical spin injection (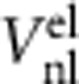) with the photo-induced signal 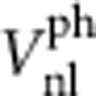. Electrical spin injection is demonstrated by applying a negative voltage to the injector (red curve, −1 μA injection current). When decreasing the applied magnetic field, starting at +150 Oe, a jump in the NL signal occurs at −50 Oe, corresponding to the magnetization reversal of the detector; the magnetization of injector and detector switch from parallel to antiparallel configuration. The corresponding change of the NL voltage 
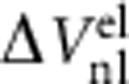
 is proportional to the spin accumulation in the GaAs channel^20^. At −100 Oe, when the narrow injector reverses its magnetization, both contacts are aligned parallel again and the NL voltage signal returns to the starting value. The same occurs at +50 and +100 Oe when sweeping the magnetic field back to +150 Oe. The small peak at zero magnetic field stems from dynamic nuclear polarization (DNP) that slightly depolarizes the spin accumulation (see Supplementary Fig. S5). The increase of the NL voltage in the antiparallel configuration for electrical spin injection corresponds to a spin up accumulation. In contrast, when reversing the bias voltage and extracting spins, 
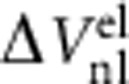
 is also reversed, corresponding to a spin down accumulation as shown by the blue and green curve for the extraction currents of 0.5 and 1 μA. From similar experiments, we estimate a current polarization of 50% (ref. 6).

If instead of electrically biasing the contact the laser spot is placed below the injector, the spin solar cell effect generates the spin accumulation, as illustrated in the upper pictogram of Fig. 3b. Corresponding spin valve curves are plotted in Fig. 3a (light blue and magenta). The absolute value of the photo-current (0.5 and 1 μA, measured separately, see Fig. 2c) reflects the absorbed laser power.

Qualitative comparison of the spin signals 
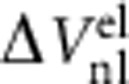
 and 
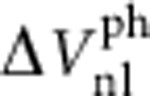
 in Fig. 3a clearly shows that the light-induced spin accumulation has the same sign as spin accumulation resulting from electrical spin extraction and the opposite sign compared with electrical spin injection. In Fig. 3b both spin valve signals 
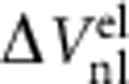
 and 
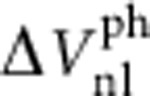
 are plotted versus the applied current *I*_el_ or photo-current *I*_ph_, respectively. The data show that the magnitude of the NL signals is similar when the photo-current matches the electrically applied current. This suggests that the laser-induced tunnelling current is, in this case, comparable to the photo-current, as it creates a similar spin accumulation. The same correlation between photo-current and generated spin accumulation is also indicated by the optical results shown in Fig. 2. Altogether, the excellent agreement of sign and amplitude of the spin accumulation with respect to the photo-current confirms the described mechanism of the spin solar cell effect.

### Spin photodiode

We now discuss the spin photodiode effect sketched in Fig. 1c. When applying a reverse bias, that is, a negative voltage to the (Ga,Mn)As contact, the depletion zone of the p-n junction increases and hence laser-induced spin extraction gets suppressed. Optically excited spin-polarized electrons in the vicinity of the p-n junction drift then from the (Ga,Mn)As conduction band to the n-GaAs channel causing spin injection. Figure 2d shows a corresponding linescan of the Kerr rotation along the n-GaAs channel while applying −10 V to the (Ga,Mn)As contact. As for the spin solar cell effect (see Fig. 2b), no spin accumulation can be observed unless the laser spot enters the contact region (shaded area). However, in contrast to the spin solar cell, this effect creates a spin up accumulation (negative signal in Fig. 2d), as expected from the explanation illustrated in Fig. 1c.

**Figure 4:**
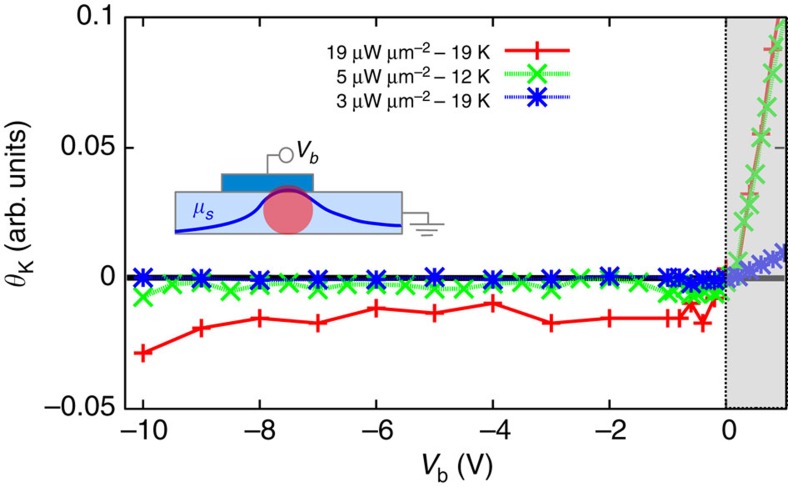
Spin photodiode effect on Sample A. Bias dependence of the spin photodiode effect (negative bias range) for low laser intensities (*λ*=816 nm). For positive voltages electrical spin extraction occurs (shaded region).

We now show that the negative bias voltage alone does not result in a spin accumulation. Sample A used here has a lower n^+^-doping compared with Sample B such that the current through the diode is largely suppressed under reverse bias (see Supplementary Fig. S8). Thus, the spin accumulation from electrical spin injection can be neglected for this sample. This is illustrated in Fig. 4 where the bias dependence of the Kerr signal is shown for three relatively low laser powers. At negative bias the spin photodiode effect appears only for larger laser power (red curve in Fig. 4), while suppressed for lower ones. This proves that the absorbed laser light and not the applied bias is responsible for generating spin accumulation. With increasing laser intensity the spin photodiode effect increases linearly as illustrated in Supplementary Fig. S9 (inset). In contrast, for positive bias voltages, electrical spin extraction occurs resulting in a visible Kerr rotation even for the lowest laser power (shaded area in Fig. 4).

## Discussion

The presented experimental verification of a spin solar cell and a spin photodiode provides an enormous potential for future spintronic applications, where additional features are added to a classical electronic device via the spin degree of freedom. The effects can be used to create a photo-sensor that converts light not only into a voltage or a current, but additionally into a spin current and a spin accumulation. To make use of the supplemental information provided by a potential spin photo-sensor, it is necessary to electrically read out the generated spin current. The inverse Spin Hall effect is a potential candidate as it is only sensitive to the spin polarization^21^. The magnetization direction of a nano-sized electrode can be electrically set via the spin transfer torque effect^22^,^23^,^24^.

Even more important is the fact that a sizeable spin accumulation can be generated using a very simple scheme. The advantage of the spin solar cell effect is the very general mechanism behind it: a potential device does not require a p-type ferromagnetic semiconductor. In fact any ferromagnetic metal could generate the same effect via a Schottky barrier. Furthermore, also a direct band gap semiconductor such as GaAs is not required, the same working principle should hold for indirect band gap semiconductors, for example, silicon or germanium. As room-temperature spin injection from various ferromagnetic metals into GaAs (refs 25, 26) or Si (ref. 27) was already achieved, all requirements for a working room-temperature spin solar cell are fulfilled. This enables a new and efficient way for the optical generation of spins in a large material class using unpolarized light, independent from optical selection rules and not limited to specific wavelengths.

## Methods

### Materials

The semiconductor p-n hetero-junction for Sample A is grown by molecular beam epitaxy on a semi-insulating GaAs(001) substrate. The spin accumulation is observed in a 1-μm thick n-GaAs channel with a doping density of 2.5 × 10^16^ cm^−3^. To achieve a narrow tunnel barrier between the 50-nm thick layer of the dilute magnetic semiconductor Ga_95_Mn_5_As, the doping density was gradually increased in a 15-nm thick transition layer, followed by a 8-nm thick layer of n^++^-GaAs with a nominal doping density of 5 × 10^18^ cm^−3^. In addition a 2-nm thick (Al,Ga)As layer was inserted between the GaAs and the (Ga,Mn)As. The Curie temperature of (Ga,Mn)As is about 60 K. Together these layers form a tunnel diode enabling tunnelling of spin-polarized electrons between the (Ga,Mn)As contact and the n-GaAs channel. The electron transport in the lateral device is confined to a 50-μm wide mesa channel with three (Ga,Mn)As contacts on top: one 4 × 50 μm injector contact and two 150 × 150 μm reference contacts at both ends of the channel with a distance of ~300 μm to the injecting contact. The device was fabricated using standard optical- and electron-beam lithography and wet etching techniques. By cleaving the sample along the [110] direction across the mesa channel, the contact area is reduced to 4 × 40 μm. In addition, the cleaving process exposes the GaAs(1–10) surface and enables direct optical access to the n-GaAs channel (see Fig. 2). Sample B consists of the same layer stack with an additional δ*-*doping in the n^++^-GaAs layer resulting in a lower interface resistance of the Esaki diode compared with Sample A (see Supplementary Figs S4 and S8). Furthermore, the width of the mesa channel is reduced to 20 μm due to cleaving and a second 0.5-μm wide (Ga,Mn)As contact is used in order to realize electrical detection in a NL voltage geometry.

### Experimental methods

The samples are mounted in a He flow cryostat, which is mounted on top of a Piezo positioning stage, for the optical and electrical measurements. This enables two-dimensional scans in the xy plane. All measurements are performed at low temperature to stay well below the Curie temperature of the (Ga,Mn)As layer of about 60 K. For the optical measurements the *z* component of the electron spin polarization (the component along [1–10]) in the n-GaAs is detected via the polar magneto-optical Kerr effect, where the spot size of the laser beam (1 μm) is similar to the thickness of the n-GaAs channel. A photon energy close to the band gap of the n-GaAs is chosen for the linearly polarized laser beam (around *λ*=816 nm at 15 K, see Supplementary Fig. S3). At this wavelength the penetration depth of the light is on the order of a few μm and hence significantly larger than the depletion zone of the n-GaAs (about 200 nm).

For the optical measurements a square-wave bias voltage alternating between zero and the target value is used while the Kerr rotation is detected synchronously by balanced photo-receivers and a lock-in technique. This ensures that the quasi-static magnetization of the ferromagnetic contacts does not contribute to the Kerr signal^17^,^18^,^19^. In addition, to eliminate any electro-optic Kerr rotation, the measurements are performed in remanence after saturating the magnetization along [1–10] and [−110], respectively. The difference in Kerr rotation *θ*_K_ of both remanent values is strictly proportional to the spin accumulation in the n-GaAs layer. Details about the modulation technique used for measuring the spin solar cell effect are described in Supplementary Fig. S2.

## Author contributions

B.E. designed the experiment and wrote the paper. M.C. prepared the samples and fabricated the devices. M.U. and D.B. realized the semiconductor film growth. M.S. assisted the electrical measurements. M.S., M.C., D.B., D.W., G.B. and C.H.B. contributed to the manuscript. G.B. and C.H.B. supervised the work.

## Additional information

**How to cite this article:** Endres, B. *et al.* Demonstration of the spin solar cell and spin photodiode effect. *Nat. Commun.* 4:2068 doi: 10.1038/ncomms3068 (2013).

## Supplementary Material

Supplementary Information Supplementary Figures S1-S11, Supplementary Note 1 and Supplementary References
