# Supplementary material for: Demonstration of the spin solar cell and spin photodiode effect
Source: Nat Commun. 2013 Jul 3;4:2068. doi: 10.1038/ncomms3068 (PMC3715846; doi:10.1038/ncomms3068)
Supplement: Supplementary Information — Supplementary Figures S1-S11, Supplementary Note 1 and Supplementary References [file ncomms3068-s1.pdf]

Supplementary information

# Demonstration of the spin solar cell and spin photodiode effect

B. Endres, M. Ciorga, M. Schmid, M. Utz,

D. Bougeard, D. Weiss, G. Bayreuther and C. H. Back

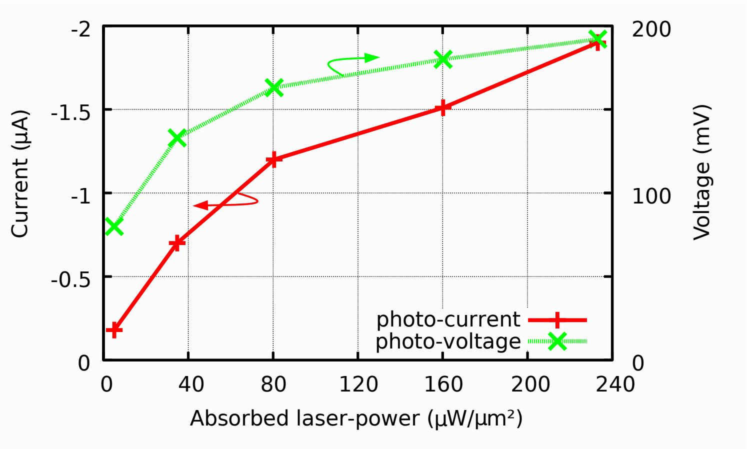


Supplementary Figure S1. **Photo-current and photo-voltage vs. laser power.** Dependence of photo-current and photo-voltage on the absorbed laser power density for 816 nm wavelength and at 15 K (Sample A). As expected, the photo-current (measured in closed circuit) shows a roughly linear dependence contrary to the photo-voltage (measured in open circuit) which already starts with a value of about 80 mV for the lowest laser power and approaches saturation with increasing power density at about 200 mV. This non-linear behavior of the photo-voltage originates from the reduction of the tunnel barrier width with increasing laser power - hence the photo-voltage is limited by the decreasing interface resistance.

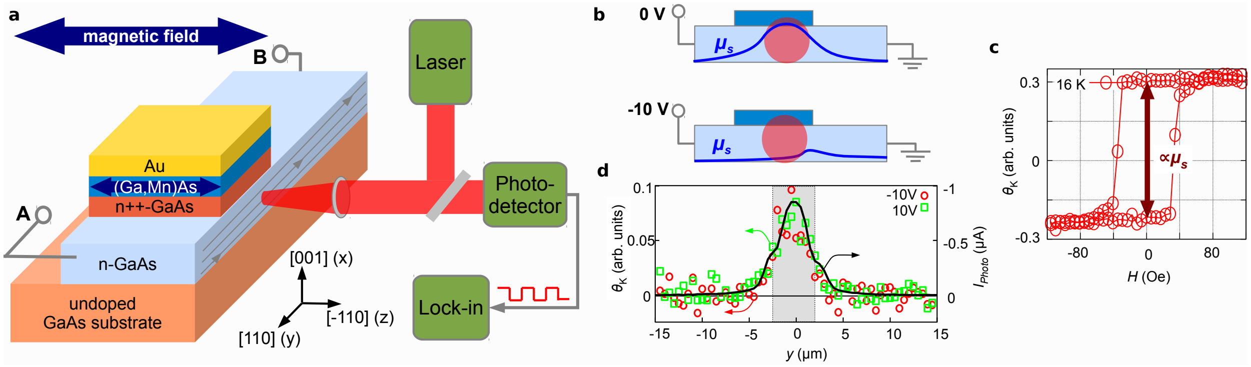


Supplementary Figure S2. **Optical detection of the spin solar cell effect.**

(a) Sample and measurement geometry for the observation of the spin solar cell effect on Sample A. The signal is modulated by applying a square-wave bias voltage $V_{AB}$, alternating between zero and ±10 V, between the two reference contacts A and B on the left and the right end of the GaAs channel.

(b) Illustration of the two modulated states for the observation of the spin solar cell effect. The situation for zero voltage is shown in in the upper panel. In contrast, when ±10 V are applied, the large electric field that builds up along the channel causes an electron drift and shifts the laser-induced spin accumulation $\mu_s = \mu_\uparrow - \mu_\downarrow$ away from the spot position as sketched in the lower panel. Thus, for large enough electric fields, the spin accumulation is almost zero at the laser spot position since the spins that are accumulated are subsequently drifting away. The Kerr rotation in this state therefore can be used as a reference signal for the case when no spin accumulation is present at the laser spot. We investigated the influence of the electric field on spin accumulation in detail in a previous publication [19].

In addition to this modulation, the measurements are performed in remanence after saturating the magnetization along [1-10] and [-110], respectively. The difference Kerr rotation signal $\theta_K$ of both remanent values is strictly proportional to the spin accumulation in the n-GaAs channel at the laser spot position. This is justified in (c).

(c) Hysteresis loop of the injected spin polarization in the n-GaAs channel by sweeping the external magnetic field along the z-direction.

(d) Kerr rotation along the n-GaAs channel showing the spin solar cell effect (green, red). Positive and negative applied voltages (-10 V, 10 V) along the channel are only used for modulation. Shaded area indicates the contact position. Due to the large interface resistance of the (Ga,Mn)As contact and a relatively large capacitance a modulation frequency of only 187 Hz was chosen for the measurements of the spin solar cell effect. For larger frequencies charging and decharging effects of the floating (Ga,Mn)As contact can be observed which correspond to electrical spin injection and extraction. However, the signal from this effect changes sign when the modulation voltage is reversed and therefore cannot be responsible for the observed Kerr rotation in (d). The position-dependent photo-current along the channel (black) was measured separately in closed circuit.

In addition, other effects like thermal spin injection due to electrical Joule heating may appear which have to be considered. First of all, it is easy to prove that no spin accumulation is generated by the relatively large voltage applied along the n-GaAs channel for the modulation of the spin solar cell effect, since the Kerr rotation signal disappears when the absorbed laser power is reduced to a probe beam (corresponding to an absorbed laser power of 5 $\mu W/\mu m^2$). Thus, thermal spin injection due to Joule heating can be ruled out.

Supplementary Figure S2. Since the observed Kerr rotation is only visible when the laser spot is at the contact region (see (d)), the spin accumulation must be created by the laser light itself. In addition the signal fits to the observed photo-current distribution that is measured when short-circuiting the junction (see black curve in (d)). Furthermore, the electrical detection of the spin accumulation on Sample B (see Fig. 3 in the main text) directly proves the equivalence of optically and electrically induced spin accumulation since the same non-local signals are observed for the same corresponding values of photo-current and electrically applied current.

Also, a temperature gradient due to the incident laser light has to be considered which could give rise to thermo-electric spin injection/extraction. In our experiment the laser beam of 1 μm diameter (FWHM) is oriented parallel to the sample plane and hits the sample at the cleaved edge consisting of 150 nm Au, 50 nm (Ga.Mn)As, a 1 μm thick n-GaAs layer and the semi-insulating GaAs substrate as illustrated in (a). The heat generated by light absorption will be carried away at the top by lateral heat conduction mainly through the Au layer, by radiation from the surface and by vertical heat conduction into the substrate. Therefore, when the laser beam is scanned in x-direction approaching the sample surface from outside a change of sign would be expected for the temperature gradient across the p-n junction at a certain beam position. Consequently, any thermal voltage created by the Seebeck effect should change sign and also any thermally-induced spin accumulation as reported in Ref. 4. Such an x-scan of the Kerr signal measuring the spin accumulation is shown in Supplementary Fig. S11b; it shows a monotonic increase of the Kerr rotation after the laser beam has moved across the sample surface. This means that a thermally induced spin accumulation does not contribute substantially to our observed signal. Furthermore, in an open-circuit situation we observe a junction voltage of about 80 mV already for a very small laser power of 5 $\mu W/\mu m^2$ (see Supplementary Fig. S1). If we tentatively assume that this voltage is mainly of thermo-electric origin, then using a Seebeck coefficient of 0.5 μV/K for GaAs/(Ga.Mn)As given by Naydenova et al. [28] a temperature difference across the junction of nearly $2 \cdot 10^5$ K would be required to generate the observed voltage. Thus, a thermo-electric origin of (photo)voltage and (photo)current can be ruled out, and this should also be true for a conceivable spin accumulation related to the spin Seebeck effect.

The influence of the absorbed laser power on the n-GaAs is shown in Supplementary Fig. S3. Altogether, this clearly shows that the observed signal unambiguously corresponds to laser-induced spin extraction - the spin solar cell effect.

In addition to the laser-induced spin extraction process also the spin-dependent reflection of optically pumped electrons (proximity effect) could be expected [29]. Since the proximity effect does not depend on doping density we tested the spin solar cell effect on a reference tunnel diode with a larger doping density (n $= 7 \cdot 10^{16}$ cm$^{-3}$) and hence a much smaller photo-voltaic effect where no significant spin polarization was observed.

Altogether, due to the excellent agreement of the optically induced signal and the photo-current with electrical spin injection (see Fig. 2 and Fig. 3 in the main text), we conclude that the spin solar cell effect must be the dominant mechanism in our device.

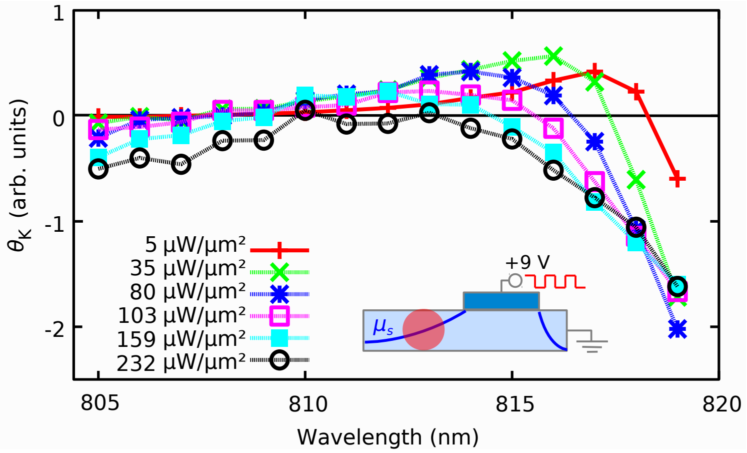


Supplementary Figure S3. **Influence of laser power on Kerr spectrum.**
Kerr-spectra for various laser intensities (absorbed power density) taken at the diffusion side next to the contact (see inset) while directly extracting spins electrically. With increasing laser power the Kerr spectrum is influenced by the additional optically generated electrons that increase the electron density in the GaAs conduction band. Therefore a quantitative dependence of the spin accumulation on laser power via the Kerr rotation is not trivial. The Kerr spectra were determined in a reference experiment on Sample A where the spin accumulation was created by direct electrical spin extraction ($V_b$ = 9 V, see inset). The spectra were observed on the diffusion side a few µm away from the (Ga,Mn)As contact in order to rule out any influence of the laser light on the contact resistance. The data clearly shows a shift to shorter wavelengths with increasing laser intensity. A shift of 1 nm corresponds to about 2 meV shift of the absorption edge or a 1 meV shift of the Fermi energy in the conduction band. Compared to the photo-voltaic effect, the increase of the Fermi energy in the n-GaAs conduction band by the optically generated electrons of a few meV is negligible. In fact, the Fermi energy increase is balanced across the whole p-n-junction in equilibrium. The additionally generated electrons and holes therefore act as an increased doping density in the p-n-junction and hence illumination will solely result in a narrower tunnel barrier. For the lowest laser power no spin solar cell effect could be observed. However, a well defined Kerr spectrum is visible as shown by the red curve so that this laser intensity can be used as a probe for spin accumulation where any other effects due to laser illumination can be ruled out.

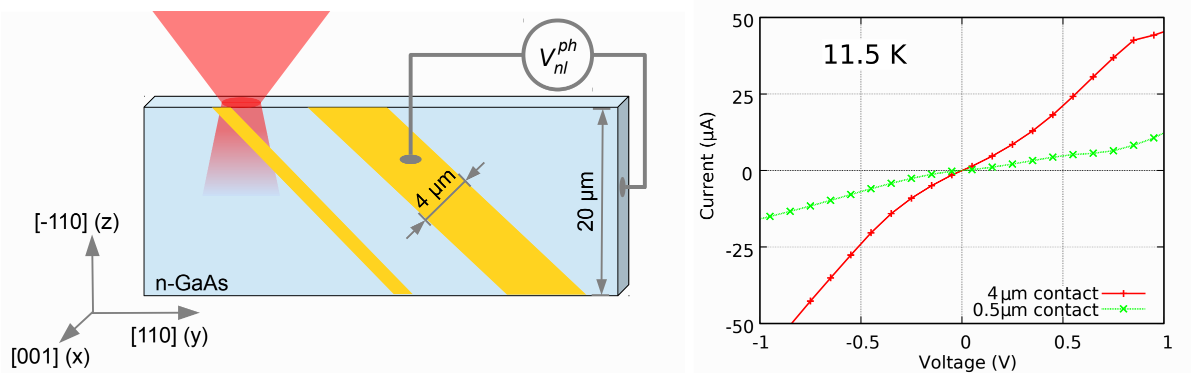


Supplementary Figure S4. **Sample B: contact geometry and $I-V$ characteristic.**
Sample B was used for the electrical detection of the generated spin accumulation. The layer structure of Sample B is similar to Sample A, only a slightly larger doping density in the GaAs at the interface to the (Ga,Mn)As exists giving rise to a more Esaki-like $I-V$ characteristic. On the left a sketch of the two (Ga,Mn)As contacts is shown (yellow, top view). The contacts were patterned along the [100] direction with an angle of 45° to the mesa channel oriented along [110]. The advantage of this geometry is on the one hand the reduction of stray-light or incoming laser-light on the detecting contact in the non-local voltage geometry (otherwise, due to the photo-voltaic effect a large offset voltage would be created even for very small light intensities). On the other hand the tilted contacts were used to check the penetration depth of the incident laser light as a function of the wavelength. On the right the $I-V$ characteristic of the 0.5 µm wide and the 4 µm wide contact is shown. In contrast to Sample A, a lower interface resistance and a more symmetric bias dependence is visible, indicating the larger doping density at the interface.

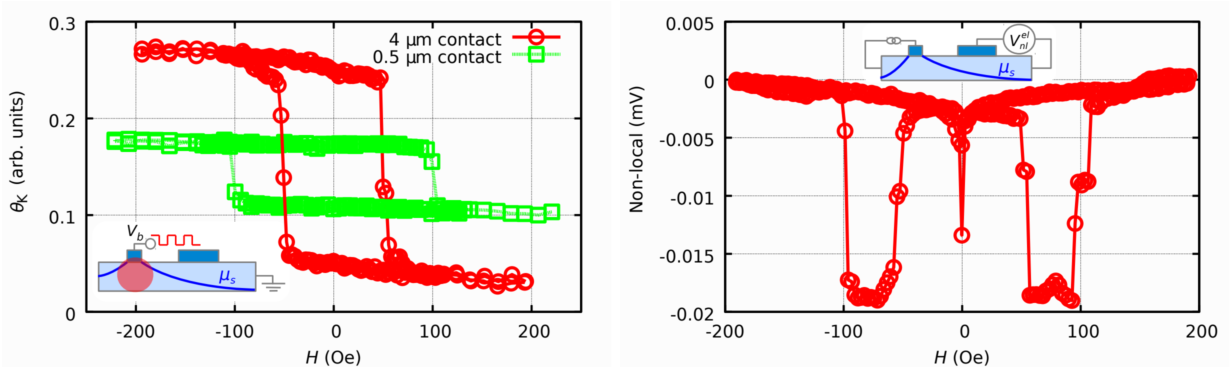


Supplementary Figure S5. **Sample B: hysteresis loops and non-local voltage signal.**
Left: Kerr rotation in the n-GaAs channel vs. applied magnetic field (z-direction) for electrical spin injection from the 0.5 µm wide and the 4 µm wide contact at 11.5 K. Right: Detection of the electrically injected spins via the non-local voltage signal (see inset) with the same magnetic field sweep at 11 K. The hysteresis curves measured magneto-optically clearly show the different switching field of the two contacts at 11.5 K when sweeping the external magnetic field along [-110] (z-direction). The switching fields of 50 and 100 Oe together with the almost rectangular switching behavior are in good agreement with the spin valve signal from the electrical detection (right).

The fact that the remanent magnetization of both contacts is not 100% shows that the [-110] direction is not the magnetic easy axis. Without external magnetic field the magnetization therefore falls into a state with a certain angle $\phi$ to the [-110] direction. This angle can be estimated by measuring the depolarization of the spin accumulation in an out-of-plane magnetic field along the x-direction (Hanle measurement). Due to the tilted magnetization the injected spins either start to precess into the laser beam axis or away from it, depending on the field direction. This must result in asymmetric Hanle curves when detected by p-MOKE which are illustrated for both contacts in Supplementary Fig. S6.

The offset-voltage which occurs in the non-local voltage signal also for electrical spin injection may originate on the one hand from a small nonuniform current that passes the detecting contact, which can be shown in 2D simulations [19], or from thermo-electric effects due to a small temperature gradient [30].

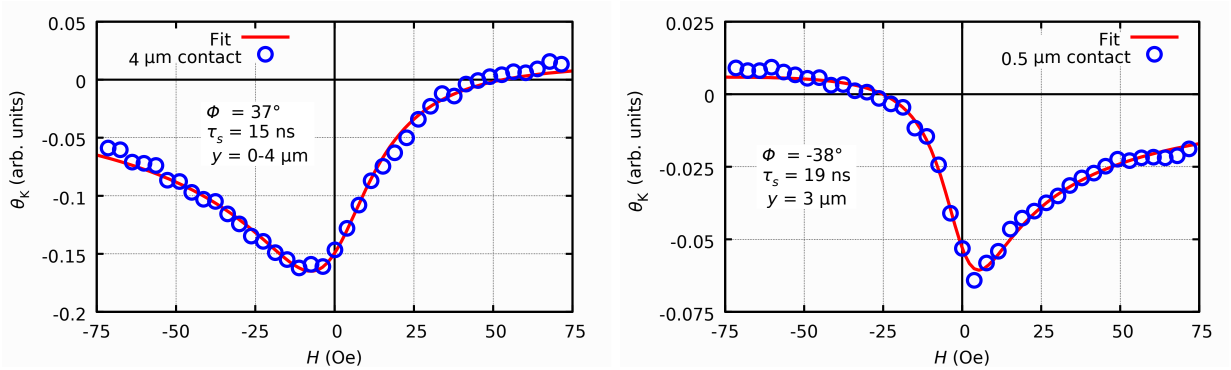


Supplementary Figure S6. **Sample B: optical detection of the Hanle effect.**

Optically detected Hanle curves for spin injection from the wide (-1 V applied) and the narrow contact (-0.5 V applied) showing the spin lifetime and the tilt $\phi$ of the magnetization with respect to the laser beam axis [-110] (z-direction). Numerical fitting the asymmetric shape of the Hanle curves as described in Supplementary Note 1 yields a magnetization tilt $\phi$ of 37° for the wide contact and -38° for the narrow contact. Thus, the magnetization of the narrow contact is oriented almost along the patterned contact direction [100] in remanence, due to the patterning induced anisotropy [31, 32] relevant for narrow contacts, and the magnetization of the wide contact is tilted in the opposite direction, almost along [010].

Consequently, when detecting the spin accumulation generated by the narrow contact electrically using the wide contact as detector, we have an angle between the spin directions of about 75°. Therefore, Hanle measurements in the non-local voltage geometry show a strong asymmetry, illustrated in Supplementary Fig. S7.

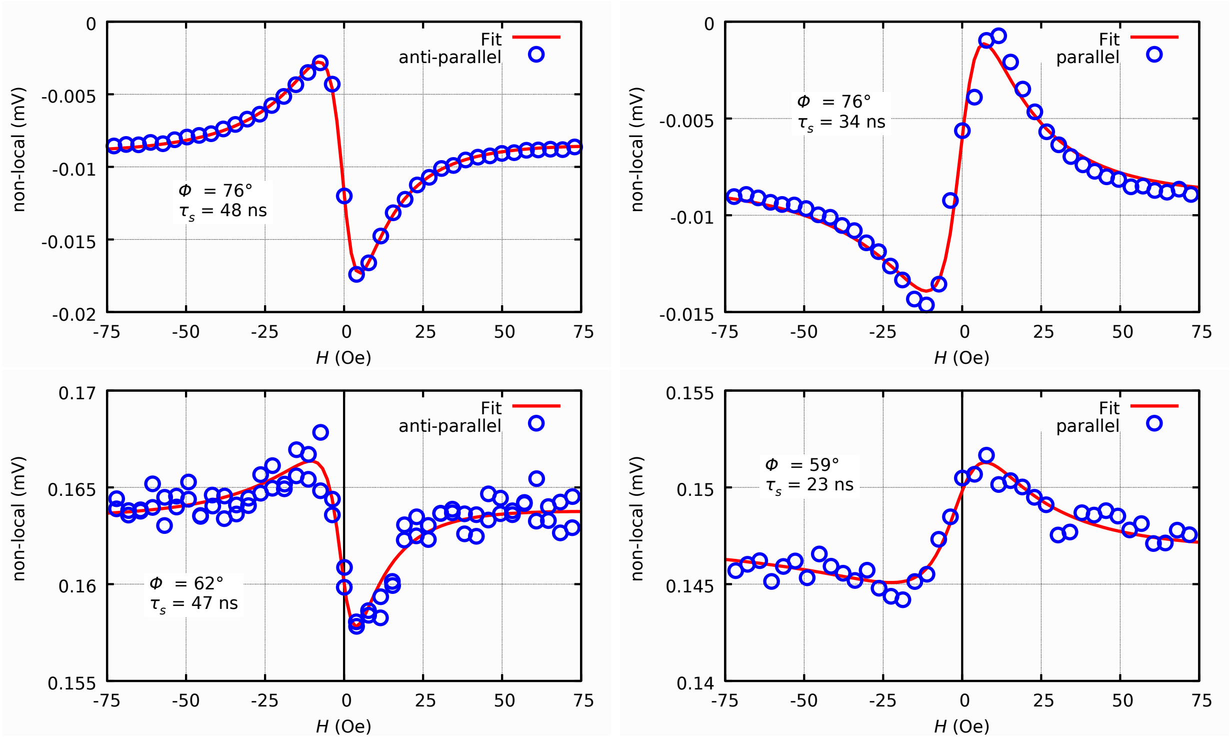


Supplementary Figure S7. **Sample B: electrical detection of the Hanle effect.**
Hanle measurements in the non-local voltage geometry for "parallel" and "anti-parallel" configuration of both contact magnetizations. Upper figures show electrical spin injection, the bottom figures show the spin solar cell effect. The extracted angle of 76° between the magnetization of both contacts matches with the results obtained by optical detection (see Supplementary Fig. S6). The bottom figures illustrate the electrically detected Hanle signal when generating the spins via the solar cell effect. The graphs again show the strong asymmetry between the contact magnetizations. The reason for the slightly smaller angle ($\sim$ 60°) probably arises from the fact that the generated spins from the solar cell effect are mostly created at the contact edge. The extracted spin lifetime from the electrical detection is usually affected by dynamic nuclear polarization (DNP) and thus overrated. From the optical detection a spin lifetime of 15-20 ns results that should be more reliable since DNP is eliminated in this measurement technique due to the fast periodic reversal of the magnetization direction [19],[33]. Details about DNP in n-GaAs, which is also responsible for the peak at zero magnetic field in the spin-valve signal (see Supplementary Fig. S5 right) are already discussed in several publications [34–38].

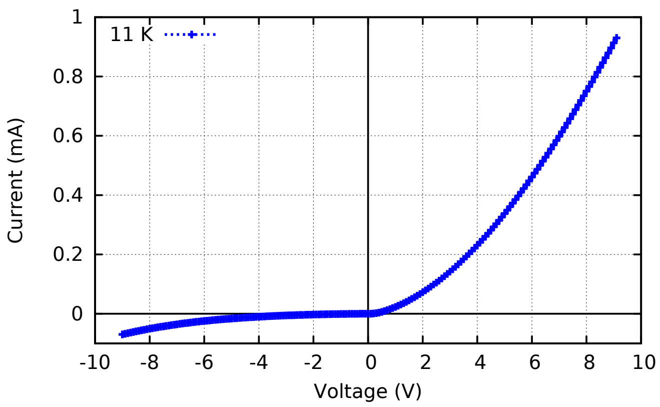


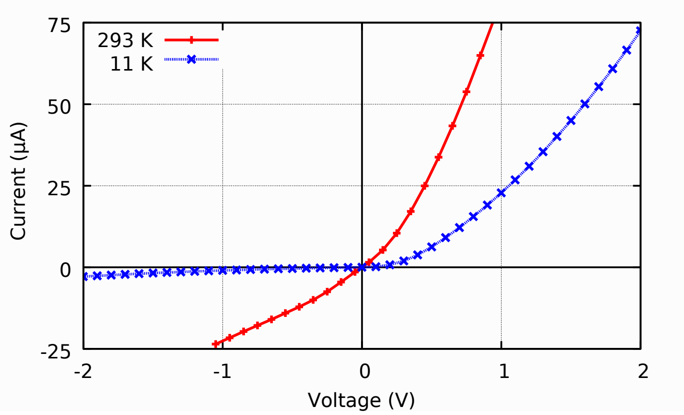


Supplementary Figure S8. **Sample A: $I-V$ characteristic of the tunnel diode.**
At low temperatures, the characteristic resembles the $I-V$ curve of an ordinary diode blocking the current in reverse direction. Electrical spin extraction up to 10 V can be demonstrated for this sample. To disable electrical spin injection in reverse bias, we adjusted the doping profile of Sample A such that the tunnel diode almost blocks the current in reverse direction. In conjunction with the decreasing spin injection efficiency with increasing negative bias voltage [6], the spin accumulation from electrical spin injection can be neglected for this sample, as shown in Fig. 4 in the main text. As a consequence the device can be used for a direct observation of the spin photodiode effect.

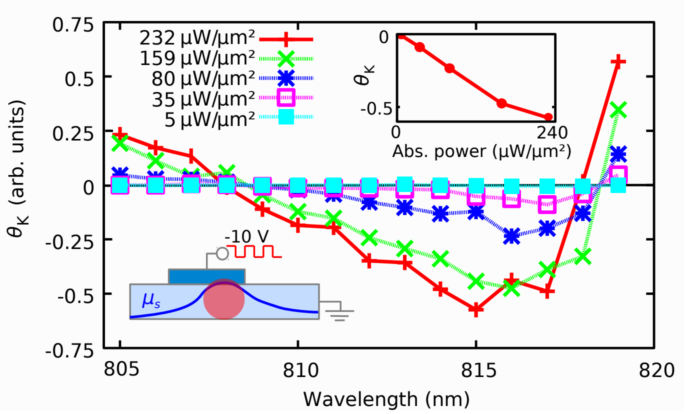


Supplementary Figure S9. **Sample A: illustration of the spin photodiode effect via Kerr spectra.** Illustration of the spin photodiode effect via the Kerr spectra for various laser intensities and an applied bias voltage of -10 V. The inset shows the maximum Kerr rotation of each spectrum versus the absorbed laser power. The estimation of the absorbed laser power density is given in Supplementary Fig. S10.

In contrast to the reference Kerr spectra shown in Supplementary Fig. S3 a reduced wavelength shift of the spectrum with increasing power density is observed. Only for the largest laser intensity the zero-crossing of the spectrum is slightly shifted to lower wavelengths by 0.5 nm. This confirms that now the spin accumulation is generated when the negative bias is applied, which also broadens the band bending in the n-GaAs and shifts the optically excited electrons away. Thus the increase of the absorption edge is less visible in this geometry. The almost linear laser power dependence of the spin photodiode effect is directly shown in the inset, where the maximum Kerr rotation is plotted as a function of laser power.

The spin photodiode effect was already proposed and calculated for a similar case, where the p-side of a nonmagnetic p-n-junction was proposed to be illuminated with circularly polarized light [16]. By applying a negative voltage to the junction, it acts as a spin photodiode, converting light into a spin-polarized charge current. In principle the effect should also be present without a negative bias, since some of the optically excited spins in the (Ga,Mn)As should reach the GaAs channel by diffusion. However, the effect seems to be rather small without negative bias since its manifestation is not observed when detecting the spin solar cell effect (see Fig. 3 in the main text).

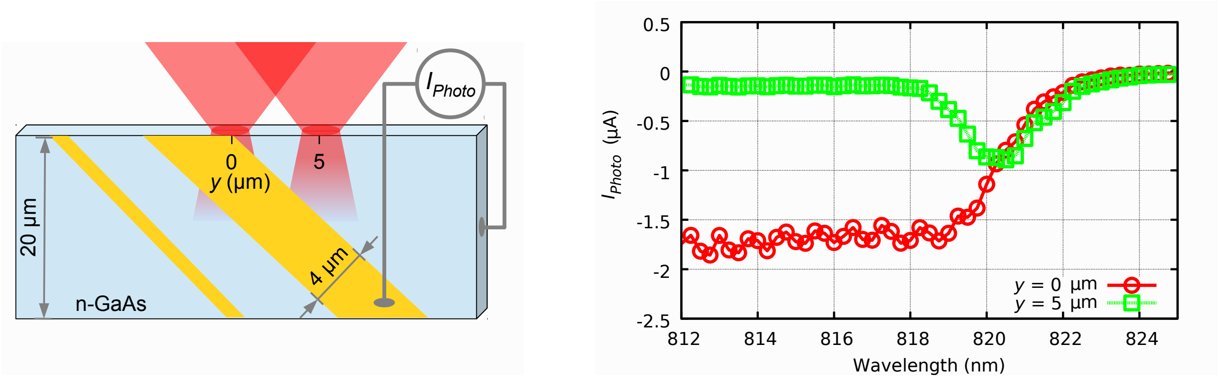


Supplementary Figure S10. **Wavelength dependence of photo-current.**

Finally we want to discuss the absorbed laser power in the n-GaAs channel. Due to the refractive index of about 3.5 for GaAs [39], 31% of the incoming laser intensity is reflected. The penetration depth of the transmitted 69% of the incident light strongly depends on the wavelength with respect to the n-GaAs band gap [40]. This is illustrated by the wavelength dependence of the photo-current on the right. The two positions of the laser spot are illustrated on the left hand side. At laser spot position $y = 0$ µm, the photo-current begins to drop above 819 nm, indicating that the penetration depth of the transmitted laser beam is exceeding the contact region. In contrast, at position $y = 5$ µm, the incoming light has to first penetrate 5 µm of GaAs before reaching the contact area. Here the photo-current is almost zero up to 818 nm and has its maximum at 820 nm. Above 822 nm the photo-current approaches zero for both cases. Thus the absorption edge lies in the energy range of 1.51 eV at 19 K. This is consistent with the observed Kerr spectra (see Supplementary Figs. S3 and S9) showing a sign reversal at about 818 nm.

In order to calculate the absorbed laser power the transmitted light is normalized to the spot size area of 1 µm diameter. The power density is then calculated by assuming that 50% of the laser intensity is located within the full width half maximum (FWHM) of the two-dimensional Gaussian spot profile (see Supplementary Fig. S11).

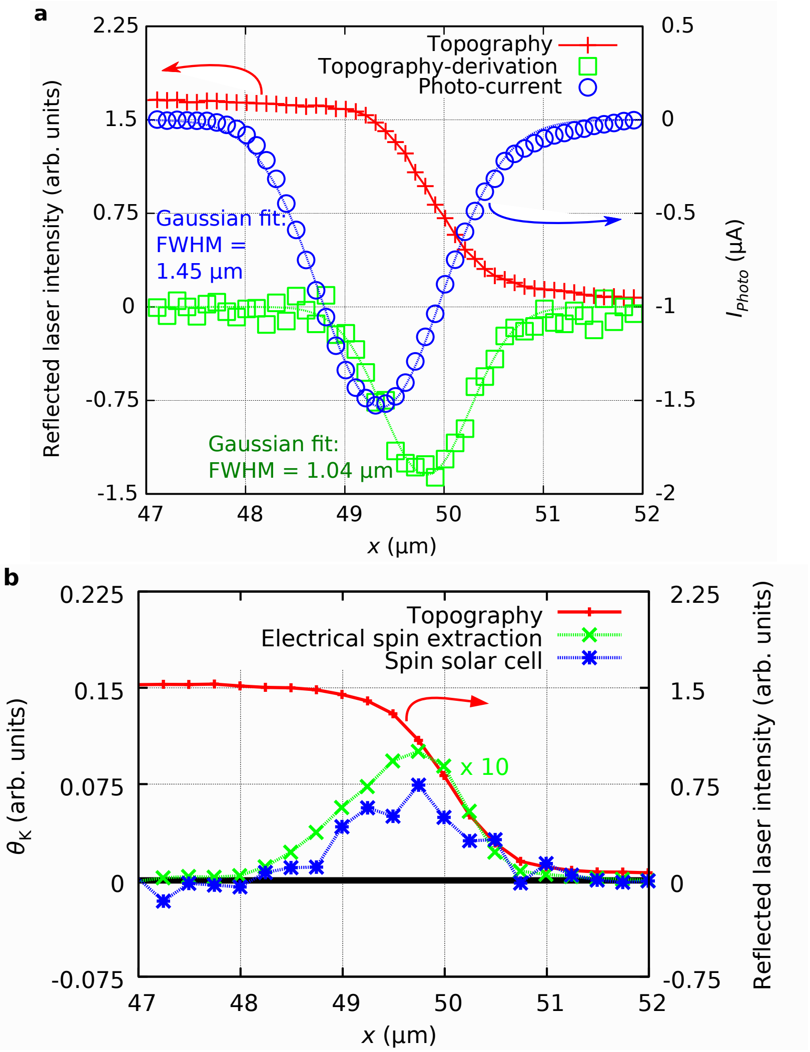


Supplementary Figure S11. **Laser beam profile and photo-current.**

(a) The red curve shows the reflected light intensity while performing a linescan across the sample edge along the x-direction at 19 K (Sample B, y = 0 µm). The derivative of this curve curve gives the laser spot profile with a FWHM of 1.04 µm (green). In addition the observed photo-current is illustrated with a FWHM of 1.45 µm (816 nm - 35 µW/µm$^2$ absorbed power density). This broadening is probably due to the penetration depth of the light and the re-emission of photons by the recombination of electron-hole pairs. The maximum of the photo-current is shifted towards the left since the active region, i.e. the band-bending region is not at the sample edge close to $x = 50$ µm, but shifted to the left due to the 150 nm thick Au layer and the 50 nm thick (Ga,Mn)As layer of the contact.

(b) Linescan across the edge of Sample A showing the Kerr rotation from the spin solar cell effect (blue) and electrical spin extraction (green, $V_b = 5$ V). The red curve shows again the reflected light intensity. Laser parameters are $\lambda = 817$ nm and 35 µW/µm$^2$ absorbed power density.

**Supplementary Note 1**

For the fitting of the Hanle data an analytical solution of the spin transport and precession in a one-dimensional model is used, derived in Ref. [41] (Eq. II.239, P.617):

$$S_z(y) = J_0 \cdot e^{-\alpha_1 y/L_s} \cdot \left[\frac{2\kappa+\alpha_1}{(2\kappa+\alpha_1)^2+\alpha_2^2}\cos\left(\frac{\alpha_2}{L_s}y\right) - \frac{\alpha_2}{(2\kappa+\alpha_1)^2+\alpha_2^2}\sin\left(\frac{\alpha_2}{L_s}y\right)\right]$$
$$\alpha_1 = \frac{1}{\sqrt{2}}\sqrt{1+\kappa^2+\sqrt{(1+\kappa^2)^2+(\omega_0\tau_s)^2}} - \kappa$$
$$\alpha_2 = \mathrm{sgn}(\omega_0)\cdot\frac{1}{\sqrt{2}}\sqrt{-1-\kappa^2+\sqrt{(1+\kappa^2)^2+(\omega_0\tau_s)^2}} \tag{S1}$$

Here, $y$ represents the distance between the detection position and the injecting contact, $\kappa$ describes the ratio of drift to diffusion, $D$ the diffusivity, $L_s = \sqrt{D\cdot\tau_s}$ the spin diffusion length, $\tau_s$ the spin lifetime and $J_0$ a scaling factor that is used as a fitting parameter. The external magnetic field is included in the Lamor frequency $\omega_0$. Since the Hanle measurements were performed on the diffusion side, no electron drift is assumed ($\kappa = 0$). A constant spin diffusion length of 6 µm is used for all curves, extracted from linescans along the GaAs channel. In addition, to take into account the width of the contact and hence all possible injection-detection distances, the fitting function is numerically integrated over the contact width.

Furthermore, the in-plane tilt of the magnetization with respect to the sensitive axis is included by using $[\cos(\phi)\cdot S_z(y) + \sin(\phi)\cdot S_y(y)]$ as fitting function with $\phi$ as additional fitting parameter, where $S_y(y)$ describes the y-component of spin polarization (see Ref. [41] Eq. II.238):

$$S_y(y) = J_0 \cdot e^{-\alpha_1 y/L_s} \cdot \left[\frac{2\kappa+\alpha_1}{(2\kappa+\alpha_1)^2+\alpha_2^2}\sin\left(\frac{\alpha_2}{L_s}y\right) + \frac{\alpha_2}{(2\kappa+\alpha_1)^2+\alpha_2^2}\cos\left(\frac{\alpha_2}{L_s}y\right)\right] \tag{S2}$$

**Supplementary References**

---


[28] Naydenova, T. et al. Diffusion thermopower of (Ga,Mn)As/GaAs tunnel junctions. *Phys. Rev. Lett.* **107,** 197201 (2011).

[29] Epstein, R. J. et al. Spontaneous spin coherence in n-GaAs produced by ferromagnetic proximity polarization. *Phys. Rev. B* **65,** 121202 (2002).

[30] Bakker, F. L., Slachter, A., Adam, J.-P., van Wees, B. J. Interplay of Peltier and Seebeck Effects in Nanoscale Nonlocal Spin Valves. *Phys. Rev. Lett.* **105,** 136601 (2010).

[31] Wenisch, J. et al. Control of Magnetic Anisotropy in (Ga,Mn)As by Lithography-Induced Strain Relaxation. *Phys. Rev. Lett.* **99,** 077201 (2007).

[32] Hoffmann, F. et al. Mapping the magnetic anisotropy in (Ga,Mn)As nanostructures. *Phys. Rev. B* **80,** 054417 (2009).

[33] Fuhrer, A., Alvarado, S. F., Salis, G., Allenspach, R. Fast electrical switching of spin injection in nonlocal spin transport devices. *Appl. Phys. Lett.* **98,** 202104 (2011).

[34] Awo-Affouda, C. et al. Contributions to Hanle lineshapes in Fe/GaAs nonlocal spin valve transport. *Appl. Phys. Lett.* **94,** 102511 (2009).

[35] Salis, G., Fuhrer, A., Alvarado, S. F. Signatures of dynamically polarized nuclear spins in all-electrical lateral spin transport devices. *Phys. Rev. B* **80,** 115332 (2009).

[36] Chan, M. K. et al. Hyperfine interactions and spin transport in ferromagnet-semiconductor heterostructures. *Phys. Rev. B* **80,** 161206 (2009).

[37] Chen, Y. S. et al. Manipulation of nuclear spin dynamics in n-GaAs using an on-chip microcoil. *J. Appl. Phys.* **109,** 016106 (2011).

[38] Shiogai, J. et al. Dynamic nuclear spin polarization in an all-semiconductor spin injection device with (Ga,Mn)As/n-GaAs spin Esaki diode. *Appl. Phys. Lett.* **101,** 212402 (2012).

[39] Blakemore, J. S. Semiconducting and other major properties of gallium arsenide. *J. Appl. Phys.* **53,** 123 (1982).

[40] Sell, D. D., Casey, H. C. Optical absorption and photoluminescence studies of thin GaAs layers in GaAs-AlxGa1-xAs double heterostructures. *J. Appl. Phys.* **45,** 800 (1974).

[41] Fabian, J., Matos-Abiague, A., Ertler, C., Stano, P., Žutić, I. Semiconductor spintronics. *Acta Physica Slovaca* **57,** 565 (2007).
